# Identification of Nine Genomic Regions of Amplification in Urothelial Carcinoma, Correlation with Stage, and Potential Prognostic and Therapeutic Value

**DOI:** 10.1371/journal.pone.0060927

**Published:** 2013-04-04

**Authors:** Yvonne Chekaluk, Chin-Lee Wu, Jonathan Rosenberg, Markus Riester, Qishan Dai, Sharron Lin, Yanan Guo, W. Scott McDougal, David J. Kwiatkowski

**Affiliations:** 1 Division of Translational Medicine, Brigham and Women's Hospital, Boston, Massachusetts, United States of America; 2 Department of Pathology, Massachusetts General Hospital, Boston, Massachusetts, United States of America; 3 Division of Genitourinary Oncology, Memorial Sloan-Kettering Cancer Center, New York, New York, United States of America; 4 Department of Biostatistics and Computational Biology, Dana-Farber Cancer Institute, and Department of Biostatistics, Harvard School of Public Health, Boston, Massachusetts, United States of America; 5 Department of Urology, Massachusetts General Hospital, Boston, Massachusetts, United States of America; Centro Nacional de Investigaciones Oncológicas (CNIO), Spain

## Abstract

We performed a genome wide analysis of 164 urothelial carcinoma samples and 27 bladder cancer cell lines to identify copy number changes associated with disease characteristics, and examined the association of amplification events with stage and grade of disease. Multiplex inversion probe (MIP) analysis, a recently developed genomic technique, was used to study 80 urothelial carcinomas to identify mutations and copy number changes. Selected amplification events were then analyzed in a validation cohort of 84 bladder cancers by multiplex ligation-dependent probe assay (MLPA). In the MIP analysis, 44 regions of significant copy number change were identified using GISTIC. Nine gene-containing regions of amplification were selected for validation in the second cohort by MLPA. Amplification events at these 9 genomic regions were found to correlate strongly with stage, being seen in only 2 of 23 (9%) Ta grade 1 or 1–2 cancers, in contrast to 31 of 61 (51%) Ta grade 3 and T2 grade 2 cancers, p<0.001. These observations suggest that analysis of genomic amplification of these 9 regions might help distinguish non-invasive from invasive urothelial carcinoma, although further study is required. Both MIP and MLPA methods perform well on formalin-fixed paraffin-embedded DNA, enhancing their potential clinical use. Furthermore several of the amplified genes identified here (ERBB2, MDM2, CCND1) are potential therapeutic targets.

## Introduction

Bladder cancer is the fourth most common cancer among men in the USA, accounting for 73,510 cases and 14,880 deaths in the US in 2011 [1]. Bladder cancer develops from the transitional cells of the mucosal urothelium and is found pathologically and clinically to occur in two mostly separate forms [2]. The first form is a non muscle-invasive tumor (stages Ta, Tis and T1), which generally has a good prognosis, but is also characterized by frequent local recurrences, requiring repeated cystoscopic evaluations. The second form is a solid, non-papillary tumor (stages T2–T4) that invades into at least the smooth muscle layer (muscularis propria), and has a high risk for metastasis. Non muscle-invasive papillary tumors growing in the lumen of the bladder constitute 70–80% of new cases each year, while invasive cases make up the remaining 20–30% at initial diagnosis [2]. Although the distinction between papillary and invasive disease is often clear on initial biopsy, the subsequent clinical behavior of each bladder cancer is uncertain, and remains a major problem in clinical management [3]. A variety of histopathologic markers have been assessed and provide some information on prognosis. However, they do not provide accurate prediction for individual patients [3]. The differences in clinical behavior as well as pathologic features suggest that there are separate oncogenic pathways for non-muscle-invasive vs. muscle-invasive bladder cancer. The vast majority of bladder cancers are urothelial carcinoma, and the same histologic type of cancer can arise throughout the urinary tract including the renal pelvis and ureters.

In patients with muscle-invasive disease, complete removal of the bladder with or without cisplatin-based neoadjuvant chemotherapy is the most commonly employed treatment approach [2]. Even with radical treatment, approximately 50% of patients develop metastatic disease, and for such patients no curative treatment exists. Furthermore, no treatment has been shown to extend survival in patients with progression following platinum-based combination chemotherapy. Thus, novel therapeutic and preventive approaches are needed for this relatively common and lethal disease.

Genetic studies of bladder cancer have a rich history, including the identification of the first oncogene, HRAS, in a bladder carcinoma cell line [4]. Subsequently, TP53 mutations were identified [5], the CDKN2A gene was shown to be a consistent target of deletion [6], and mutations were found in both the TSC1 and FGFR3 genes in bladder cancer [7], [8], as well as many other genetic changes. In addition, comparative genomic hybridization has been used extensively to identify regions of chromosomal gain and loss in bladder cancer with identification of many consistent changes of potential importance in tumor development although the resolution of these technologies was limited [6], [9]–[13].

Here, we report genomic analysis of 164 urothelial carcinoma samples and 27 bladder cancer cell lines. To assess genomic copy number changes genome-wide, we performed molecular inversion probe (MIP) analysis, which works well on formalin-fixed paraffin-embedded (FFPE) tumor specimens. We identified 44 regions of copy number change in a discovery cohort of 80 urothelial carcinoma samples, and then focused on 9 genomic regions showing significant and relatively common amplification of genes that may function as ‘driver’ events for urothelial carcinoma development. We validated these regions in a replication design by analysis of a set of bladder cancer cell lines, on nearly half of the original samples, and on a validation cohort of 84 FFPE bladder cancer samples. We found that genomic copy number changes were significantly more common in Ta grade 3 and higher stage/grade tumors than in stage Ta grades 1 and 1–2 tumors. These observations suggest that analysis of these genomic regions might be a useful diagnostic tool to determine the invasive potential of bladder cancer. In addition, the genes in some of these amplified regions are potential therapeutic targets.

## Materials and Methods

### Human urothelial carcinoma specimens and cell lines

Urothelial carcinoma specimens that had been formalin fixed and embedded in paraffin using standard techniques were obtained from the Pathology archives of the Massachusetts General Hospital (Table S1). Forty of these samples had portions that were rapidly frozen at −80°C as well. Written consent was obtained from each patient for this study on a protocol that was approved by the hospital's institutional review board, “Partners Human Research Committee”. Bladder cancer staging was performed according to the current AJCC guidelines [14]. Grade was determined according to the 1973 WHO bladder cancer guidelines [2].

Twenty-four bladder cancer cell lines were obtained from the stocks of the Translational Urology Research Lab at Massachusetts General Hospital, MA. Two other bladder cancer cell lines were generously provided by Margaret A. Knowles (St James's University Hospital, UK) [15], and one was obtained from a German cancer cell line bank. These 27 bladder cancer cell lines were maintained in Dulbecco's Modified Eagle Medium (Cellgro, Manassas, VA) supplemented with 10% Fetal Bovine Serum and 1% penicillin-streptomycin-amphotericin B (Life Technologies, Carlsbad, CA), in an incubator at 37°C in 5% CO2. All 27 cell lines were subject to microsatellite fingerprinting which confirmed that they were unique.

Anonymized discard normal human blood samples were obtained from the clinical laboratory at Brigham and Women's Hospital on a protocol that was approved by the hospital's institutional review board, “Partners Human Research Committee”. These were used to prepare control normal DNA.

### DNA extraction

DNA was extracted from formalin-fixed paraffin embedded (FFPE) samples using the BiOstic FFPE Tissue DNA isolation Kit (MO BIO Laboratories, Inc., Carlsbad, CA). DNA was extracted from blood using the QIAGEN DNeasy Blood and Tissue kit. DNA was extracted from cell lines and frozen cancer specimens using the Puregene DNA Purification kit following the protocol for 1–2 million cultured cells. DNA concentrations were determined by nanodrop and confirmed by agarose gel electrophoresis.

### Molecular Inversion Probe (MIP) Assay

A molecular inversion probe (MIP) assay examining 330,000 single nucleotide polymorphisms (SNPs) and 412 cancer gene mutations in 46 cancer-related genes (OncoScan) was performed with the assistance of Affymetrix in Santa Clara, California [16]. The SNPs had an average intermarker distance of 3 kb for the 150,000 genic probes, and 9 kb for the non-genic probes. The 412 cancer gene mutations are listed in Table S2. The raw data from this analysis (CEL files) has been put in the GEO archive (GSE44323).

### Biostatistical Software Tools

The raw MIP intensity data provided by Affymetrix was loaded and analyzed using Nexus Copy Number v6.0 (BioDiscovery Inc., El Sequendo, CA). Data was normalized using the SNP-FASST2 segmentation algorithm. Normalized probe intensity and allele ratio data were visualized in Nexus v6.0. The quality of the copy number data from each sample was assessed by measuring the Median of Absolute Pairwise Distribution (MAPD). The absolute pairwise difference (APD) is calculated as the log_2_ value of the ratio of CN intensity values for each adjacent pair of probes, across the entire set of 330,000 probes.

Normalized copy number data was then segmented using the GLAD algorithm available in GenePattern 3.3.3 [17]. Recurrent copy number alterations were identified using Genomic Identification of Significant Targets in Cancer (GISTIC) [18], implemented in both GenePattern 3.3.3 and in Nexus v6.0. GISTIC identifies regions of the genome that are significantly amplified or deleted across a set of cancer samples. Each amplification or deletion event is assigned a G-score that considers the amplitude as well as the frequency of occurrence among the sample set. False Discovery Rate q-values are then calculated for each region of gain or loss. Regions with q-values <0.1 were considered significant. Genomic coordinates used in the Tables are all from human build hg18.

### Multiplex Ligation-Dependent Probe Amplification (MLPA)

MLPA probe sets targeting 16 genes in 9 genomic regions were designed following methods we have used previously [19] (Table S4). Individual probe oligonucleotides (size range 45–84 nt) were synthesized by Integrated Device Technology (IDT, Coralville, IA). MLPA assays were performed on 100–150 ng genomic DNA samples using the MRC Holland Salsa MLPA EK5 reagent kit (MRC Holland, Amsterdam, the Netherlands). MLPA products were separated by capillary electrophoresis on the ABI 3130, and light intensity reflecting fluorescence was captured according size of the fragment, in comparison to Rox 500 size standards. Ten to 24 DNA samples were subject to MLPA analysis in each run. For analysis of control blood DNA and bladder cancer cell line DNAs, the blood DNA samples were used as controls for normalization, as described [19]. Because the amplification patterns of FFPE DNA by MLPA were different from those seen with blood or cell line DNA, a different method was used for normalization of FFPE DNA samples. Within each run, peak heights were initially normalized using all samples analyzed. Samples with no amplification were then identified and used as normalization controls for that particular MLPA run. In practice, several FFPE DNA samples for which there was a large amount of DNA were run repeatedly on different runs and typically served as the controls for several runs. To determine the reproducibility of the assay, replicate analyses of samples in different MLPA runs were compared for each probe value by calculating the coefficient of variation. The coefficient of variation was calculated as the standard deviation of a pair of measurements divided by the mean of those two measurements. A coefficient of variation of <10% was considered a robust assay.

### Sanger Sequencing

Sanger sequencing was performed on PCR products by standard methods in the BWH DNA Sequencing Core Facility. Sequencing traces were viewed and analyzed using FinchTV v1.4.0.

### Statistical methods

The Fisher exact test was used for analysis of categorical data, and computed in Prism (v4.0a, GraphPad Software, Inc.).

## Results

### Urothelial carcinoma patient characteristics

One hundred sixty-four urothelial carcinoma specimens prepared in paraffin were used to obtain FFPE DNA for this analysis, and were divided into two cohorts (Table 1, S1). The discovery cohort of 80 samples included cystectomy, nephroureterectomy, and transurethral resection specimens, and had tumor stages ranging from Ta to T4 (Table 1), but were nearly all T1 or higher stage to permit robust detection of mutations associated with invasive urothelial carcinoma. The validation cohort of 84 samples were obtained exclusively by transurethral resection, were a sequential series and consisted of tumor stages Ta–T2 (Table 1). Full clinical and demographic information on these patients and cancers is given in Table S1.

**Table 1:** Stage and grade information for 164 urothelial carcinoma samples.

Discovery cohort		
stage grade	#	%
Ta g1,2	3	4%
T1 g2	3	4%
T1 g3	36	49%
T2 g2	1	1%
T2 g3	6	8%
T3 g3	17	23%
T4 g2	2	3%
T4 g3	12	16%
total	80	
Validation cohort		
stage grade	#	%
Ta g1	13	15%
Ta g1–2	10	12%
Ta g3	20	24%
T2 g3	41	49%
total	84	

Upper, 80 samples in the discovery cohort analyzed by MIP. Bottom, 84 samples in the validation cohort analyzed by MLPA.

### Molecular Inversion Probe (MIP) genetic mutational analysis

We used MIP analysis [16], to examine both genomic copy number and mutation at 412 potential sites in 46 cancer-related genes (Table S2) on the discovery cohort of 80 urothelial carcinoma FFPE samples. The wide range of grades and stages in this cohort were selected by design to permit analysis of the broad spectrum of urothelial carcinoma. First, we analyzed the mutations identified by the MIP analysis.

Thirty-two mutations in 7 genes were identified in 28 urothelial carcinoma samples by Affymetrix criteria (scores≥9.0, and <50) as being probable mutations in the 80 urothelial carcinoma FFPE samples. To validate these findings, we performed Sanger sequencing for each mutation-sample pair, and confirmed 20 of the 28 mutations that were called in the MIP analysis (Table 2). Of those that failed to validate, the majority had Affymetrix mutation scores <10.0 or >25. Twenty of 22 (91%) mutation calls with scores between 10.0 and 25 validated by Sanger sequencing.

**Table 2:** Mutations in the discovery cohort of 80 urothelial carcinoma samples identified by MIP analysis and validated by Sanger sequencing.

Gene	Nucleotide	Amino acid	#^1^	Stages^2^
ATM	2572T>C	F858L	3	T1, T1, T4
FBXW7	1393C>T	R465C	1	T1
FGFR3	1118A>G	Y373C	4	T1, T1, T1, T1
HRAS	34G>A	G12S	1	T1
KRAS	35G>A	G12D	1	T1
PIK3CA	1624G>A	E542K	4	T1, T1, T3, T4
TP53	742C>T	R248W	1	T1
TP53	853G>A	E285K	4	T1, T3, T3, T3
TP53	818G>A	R273H	1	T3

1 Number of different samples with this mutation.

2 Stage of the urothelial carcinoma samples with mutation.

### Molecular Inversion Probe (MIP) genomic copy number variation analysis

We then focused on analysis of the genomic copy number information provided by the MIP analysis. Seven (9%) urothelial carcinoma MIP results were excluded from genomic copy number analysis due to having a MAPD score >1.5, reflecting a high variance in probe to probe measurement (see Methods for details). The median MAPD for the remaining 73 samples was 0.50. Nexus v6.0 (Bio-Discovery) was used to visualize both copy number and allele ratio information for the 330,000 SNPs across the genome (Figure S1).

**Figure 1:**
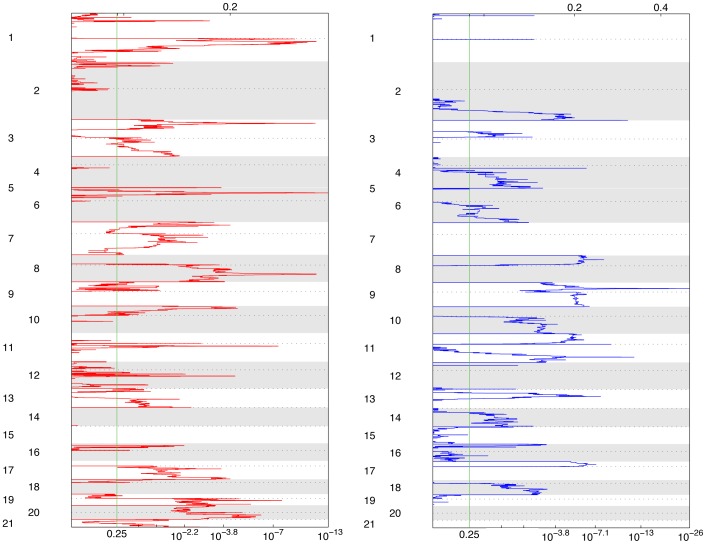
GISTIC plot of genomic regions with CN gain or loss from the MIP analysis on 73 urothelial carcinoma specimens. The 21 autosomes are shown on the y axis, and q values indicating statistical significance from the GISTIC analysis are plotted on the x axis for regions of copy number gain (red at left) and loss (blue at right).

GISTIC [18] was used to identify regions of significant CN gain or loss in the 73 urothelial carcinoma samples (Figure 1). Forty-four chromosomal regions showed a statistically significant CN loss or gain, with q-value <0.1 (Table S3). These 44 chromosomal regions were compared with a large set of similar CN gain and loss regions available at the tumorscape web-site for other cancers (http://www.broadinstitute.org/tumorscape), and also examined using the Integrated Genome Viewer (IGV, see Methods). Fourteen regions of gain or loss which did not contain protein-coding genes were not considered further, reducing the number of regions to 16 regions of gain and 14 regions of loss.

We then chose to focus on the 16 regions of amplification since such regions often contain genes that are ‘drivers’ of cancer development, and may be amenable to specific therapeutic targeting. Manual review of high resolution copy number and allele frequency graphs was performed in both Nexus (Figure S2) and IGV, and nine regions were identified in which there was high level amplification with at least 3 of the 73 urothelial carcinoma samples showing a copy number >5 in the region (Table 3). (Note that these values are not corrected for stromal cell contamination in these samples, so that there are likely more samples with true cancer amplification to >5 copies.) For many of these regions, the amplification target was known from previous studies in bladder and other cancers. However, the identity of the gene on chromosome 1q23.3 was not well-defined from previous work, and for chromosome 6p22.3, there were two candidate genes, *E2F3* and *SOX4*. Hence we chose to analyze 16 distinct genes (Table 4) in a replication study to validate the MIP findings, and examine the possible association of amplification of these regions with bladder cancer stage and grade.

**Table 3:** Identification of nine genomic regions with high level amplification seen in at least 3 of 73 urothelial carcinoma samples in the discovery cohort by MIP analysis.

chromosome	1p34.2	1q23.3	3p25.2	6p22.3	8p11.2	8q22.2	11q13.2	12q15	17q12
q value	6.47E-04	2.29E-12	3.02E-12	5.26E-14	5.28E-02	2.29E-12	3.01E-08	3.57E-05	1.89E-04
chr region	chr1:39.5–41.0 Mb	chr1:159.1–159.7 Mb	chr3:12.2–12.5 Mb	chr6:21.6–22.0 Mb	chr8:42.34–42.36 Mb	chr8:101.2–103.1 Mb	chr11:68.6–69.6 Mb	chr12:67.3–68.3 Mb	chr17:34.9–35.2 Mb
*copy number	16.0, 6.5, 6.1	12.1, 9.2, 8.6	18.0, 8.0,7.0	17.2, 15.6, 9.1	10.3, 7.7, 5.2	10.5, 7.6, 5.7	17.5, 9.8, 8.7	16.0, 12.8, 10.8	16,0, 5.5, 5.5
genes	MYCL1	TSTD1	PPARG	SOX4	POLB	YWHAZ	CCND1	MDM2	ERBB2
	hsa-mir-30c-1	PVRL4	SYN2	E2F3	MYST3	POLR2K	FGF3	CPM	GRB7
	BMP8B	NIT1	TSEN2		AP3M2	SPAG1	FGF4	LYZ	NEUROD2
	COL9A2	DEDD			PLAT	RNF19A	FGF19	YEATS4	PNMT
	NFYC	UHMK1			IKBKB	PABPC1	MYEOV	CCT2	TCAP
	PPT1	DDR2				ZNF706	TMEM16A	FRS2	STARD3
	RLF	NUF2				GRHL2	ORAOV1	CPSF6	IKZF3
	PABPC4	APOA2				NCALD		SLC35E3	PPP1R1B
	RIMS3	FCER1G				ANKRD46		NUP107	C17orf37
	ZMPSTE24	FCGR2A				SNX31		BEST3	PERLD1
	PPIE	MPZ				FBXO43		LRRC10	
	CAP1	NDUFS2							
	MACF1	PFDN2							
	HEYL	PPOX							
	HPCAL4	SDHC							
	TRIT1	USF1							
	OXCT2	B4GALT3							
	SMAP2	ADAMTS4							
	C1orf176	NR1I3							
	ZNF643	USP21							
	NT5C1A	F11R							
	MFSD2	UFC1							
	TMCO2	ITLN1							
	ZNF684	TOMM40L							
	ZNF642	KLHDC9							
	BMP8A	ITLN2							
	KIAA0754	ARHGAP30							
		C1orf192							
		LOC642502							
		PCP4L1							
		LOC100134860							

* copy number values of the 3 urothelial carcinoma samples with the largest amplification of the region (largest to smallest).

**Table 4:** Chromosomal regions and genes analyzed by MLPA.

chromosome	1p34.2	1q23.3	3p25.2	6p22.3	8p11.2	8q22.2	11q13.2	12q15	17q12
q value	6.47E-04	2.29E-12	3.02E-12	5.26E-14	5.28E-02	2.29E-12	3.01E-08	3.57E-05	1.89E-04
chr region	chr1:39.5-41.0 Mb	chr1:159.1-159.7 Mb	chr3:12.2-12.5 Mb	chr6:21.6-22.0 Mb	chr8:42.34-42.36 Mb	chr8:101.2-103.1 Mb	chr11:68.6-69.6 Mb	chr12:67.3-68.3 Mb	chr17:34.9-35.2 Mb
genes	MYCL1	TSTD1	PPARG	SOX4	POLB	YWHAZ	CCND1	MDM2	ERBB2
		PVRL4		E2F3					
		NIT1							
		DEDD							
		UHMK1							
		DDR2							
		NUF2							

### Multiplex Ligation-dependent Probe Assay (MLPA) in bladder cancer cell lines

To replicate these findings, and to generate an assay more easily applied to routine clinical samples, we generated a set of MLPA probes for each of the 16 genes in Table 3 (Table S4). The MLPA assay appeared to work robustly, with the exception of a single control probe set which was subsequently dropped from consideration, and was applied to a set of control blood DNA samples and a set of bladder cancer cell lines (Figure S3). Replicate analyses performed on four blood DNA samples indicated that the coefficient of variation for copy number determined by MLPA analysis was a median of 3.64% and a mean of 4.40%, indicating a relatively low level of variation in this assay. Replicate analyses of copy number performed on 25 bladder cancer cell line DNA preparations indicated that the coefficient of variation was a median of 6.57% and a mean of 7.77% (two samples could not be replicated due to insufficient DNA). This is still a relatively low level of variation, but is higher than blood DNA samples, likely due to the presence of amplified regions in many of the bladder cancer cell lines, which diminishes the size of non-amplified products, increasing the variance in duplicate measurements.

Eleven of 27 (41%) bladder cancer cell lines analyzed by the MLPA assay had amplification of one or more of the 16 genes assayed (Table 5), while the remainder had no amplification among the tested genes, considering amplification to be 4 or more copies. Six (22%) cell lines showed amplification of CCND1, while 5 each showed amplification of some or all of the genes on 1q23.3 and of E2F3-SOX4. No cell line had amplification of POLB or ERBB2. These findings validate the original MIP analysis as 7 of the 9 genomic regions of amplification seen by MIP analysis were also seen in these bladder cancer cell lines.

**Table 5:** MLPA analysis of amplification in bladder cancer cell lines.

Chromosome:	1p34.2	1q23.3	1q23.3	1q23.3	1q23.3	1q23.3	1q23.3	1q23.3	3p25.2	6p22.3	6p22.3	8p11.2	8q22.3	11q13.3	12q15	17q12
	MYCL1	TSTD1	PVRL4	NIT1	DEDD	UHMK1	DDR2	NUF2	PPARG	E2F3	SOX4	POLB	YWHAZ	CCND1	MDM2	ERBB2
UMUC1														10.1		
UMUC7	11.2	6.9	7.4	6.7	7.3	8.2	7.3	7.3			5.2					
UMUC10		5.7	6.1	5.7	5.8					8.9	14.5					
UMUC11														10.3	6.6	
UMUC17														4.2		
HT1376	5.1	5.1	5.8	5.1	5.6	5.4	4.5	4.6		4.9	9.3					
SCABER		4.3	4.9	4.4	4.6								5.6	5.7		
HT1197											4.1					
5637									10.0	11.3	22.4			4.2		
BL13															16.0	
BL138		4.4	4.8	4.6	4.6	4.6	4.3	4.4						12.7	4.1	

Genomic copy number is shown only when values are ≥4.0. bladder cancer cell lines had no amplification and are not listed above: J82, 639V, MGH-U1, MGH-U3, MGH-U4, MGH-U5, RT4, T24, 253J, 647V, UMUC15, BL17, UMUC3, UMUC6, HCV29, 97–1.

### MLPA validation of MIP analysis

To further validate the copy number findings made in the MIP analysis, we performed MLPA analysis of DNA prepared from parallel fresh frozen samples from 39 of the cancers analyzed in the first cohort of 80 FFPE samples. We found that 343 of 351 (98%) of the chromosomal regions analyzed on the paired samples by the two methods showed concordance in detection of amplification or lack of amplification, assessed as CN > 4. Furthermore of the 27 chromosomal regions of amplification detected by either MIP or MLPA analysis in individual samples, 19 (70%) showed concordance by the two methods of analysis. Concordant results were seen for amplification in 8 of the 9 genomic regions analyzed for one or more cancers. We suspect that the lack of concordance seen for some samples may reflect tumor heterogeneity with significant differences in gene amplification events seen in different samples of the same cancer, or possibly differences in tumor content in the two samples. Overall, we take these findings as strong validation of the MIP analysis method.

### MLPA analysis of the validation cohort and comparison with stage

We then performed a replication analysis assessing amplification of these 9 genomic regions and 16 genes on the validation cohort of 84 FFPE bladder cancer DNA samples. Furthermore, we examined the possibility that genomic amplification of these regions would be associated with stage of disease, and thus might provide a potential prognostic measure for clinical use. Since most clinical bladder cancer specimens on which initial treatment decisions are based are derived from transurethral resection, we analyzed FFPE samples obtained only by that means, and that ranged in stage from Ta grade 1 through T2 grade 3.

To demonstrate the reproducibility of the MLPA assay on FFPE DNA samples, replicate analyses were performed on 26 of the 84 FFPE samples, all of those for which DNA was available. On these samples the coefficient of variation for replicate analyses had a median of 5.66% and an average of 6.75%, indicating that the MLPA assay was reproducible and robust.

Considering amplification to be 4 or more copies, 33 of 84 (39%) samples analyzed showed amplification of one or more genomic regions (Table 6). Similar to the findings with the bladder cancer cell lines, CCND1, the chromosome 1q23.3 region, and E2F3-SOX4 were the most commonly amplified, seen in 11 (13%), 8 (10%), and 12 (14%) samples, respectively.

**Table 6:** MLPA analysis of amplification in the validation cohort of 84 bladder cancer FFPE samples.

Chromosome:	1p34.2	1q23.3	1q23.3	1q23.3	1q23.3	1q23.3	1q23.3	1q23.3	3p25.2	6p22.3	6p22.3	8p11.2	8q22.3	11q13.3	12q15	17q12
	MYCL1	TSTD1	PVRL4	NIT1	DEDD	UHMK1	DDR2	NUF2	PPARG	E2F3	SOX4	POLB	YWHAZ	CCND1	MDM2	ERBB2
Ta/grade 1–2														6.9		
Ta/grade 1–2	4.3		4.2											5.2		5.1
Ta/grade 3	4.3										4.6			4.4		
Ta/grade 3													4.5	34.7	14.8	
Ta/grade 3		4.6	4.7	4.5	4.6		4.4	4.3			4.2					
Ta/grade 3														26.0	7.7	
Ta/grade 3														4.2		
Ta/grade 3										5.3	6.5					
Ta/grade 3										10.9	4.6					
Ta/grade 3															4.8	
Ta/grade 3		4.0	4.5	4.2	5.2	4.4	4.3	7.2								
Ta/grade 3												5.1				
Ta/grade 3										5.0		5.4				
T2/grade 3									8.0			6.1	5.0	21.9	10.8	
T2/grade3									5.1							
T2/grade 3							5.0	4.2					5.0			
T2/grade 3		7.7	5.9	6.3	5.9		6.3	5.3								
T2/grade 3		4.1	4.2	4.5												
T2/grade 3											7.4					
T2/grade 3		5.6	4.9	4.4	4.9			4.2						40.7	12.0	
T2/grade 3										6.7	10.6					
T2/grade 3											5.7		4.4			
T2/grade 3														19.8		
T2/grade 3		5.3	4.8	4.5	4.2								7.3			
T2/grade 3	4.1									7.3	11.1					
T2/grade 3									4.1							
T2/grade 3												4.7				
T2/grade 3									6.1							
T2/grade 3										6.6	5.0					
T2/grade 3														4.1		
T2/grade 3																4.5
T2/grade 3											10.1			4.2	16.4	
T2/grade 3										5.1	7.8				26.5	

Genomic copy number is shown only when values were ≥4.0.

51 samples are not listed, as they had no amplification events detected (all CN<4.0). The stage and grade distribution of these samples was: 13 Ta grade 1; 8 Ta grade 1–2; 9 Ta grade 3; 21 T2 grade 3.

The frequency of any amplification event was strongly correlated with tumor stage and grade (Table 7). Amplification events were seen in only 2 of 23 (9%) Ta grade 1 or 1–2 cancers. In contrast, amplifications were seen in 11 of 20 (55%) Ta grade 3 cancers, and in 20 of 41 (49%) T2 grade 2 cancers. Comparison of the frequency of amplification among these three groups is highly significant with p = 0.0020 and 0.0011, comparing the first group with each of the second two groups (Fisher's exact test). SOX4 was the only individual marker which showed amplification at a significantly higher rate in Ta grade 3+T2 grade 2 cancers (11 of 61) than in Ta grade 1 or 1–2 cancers (0 of 23, p = 0.03). Examination of the frequency of amplification of these 9 genomic regions in the discovery cohort of 73 samples analyzed by MIP assay showed a similar trend, but the results were not statistically significant due to the small number of Ta tumors in that cohort. None of 3 Ta grade 1 or 2 samples showed amplification, and 31 (44%) of 70 T1–T4 samples showed amplification of one or more of the 9 genomic regions. Since the low frequency of copy number variation in the Ta grade 1 and 1–2 samples might be explained by presence of normal tissue rather than bladder cancer in those specimens, we examined them for mutations in FGFR3. Ten of 11 samples examined (4 grade 1 and 7 grade 1–2) had mutations in FGFR3: 1 had R248C, 8 had S249C, and 1 had Y373C, consistent with previous studies of early stage bladder cancer [20], [21].

**Table 7:** Summary of MLPA findings in FFPE bladder cancer samples according to stage.

		total #	any CN≥4.0	%
group 1	Ta grade 1	13	0	0%
	Ta grade1–2	10	2	20%
group 2	Ta grade 3	20	11	55%
group 3	T2 grade 3	41	20	49%
	total	84	33	39%

Groups 1, 2, and 3 have a statistically significant difference in the frequency of any amplification event, with p = 0.0020 comparing groups 1 and 2, and p = 0.0011 comparing groups 1 and 3. P is not significant comparing group 2 with group 3. Fisher exact test.

## Discussion

In this study, we used an innovative methodology, MIP analysis, to examine both a set of 412 mutations and to perform copy number analysis across the genome using 330,000 SNP probes. The MIP procedure enables analysis of FFPE DNA, the most commonly available clinical material. Most mutations identified here were in the 12 most commonly mutated genes reported by the Catalogue of Somatic Mutations in Cancer (COSMIC) in bladder cancer [22]. However, we also identified mutations in two other genes not reported in COSMIC for bladder cancer, ATM and FBXW7. Relatively few mutations in FGFR3 were seen in this cohort, four Y373C (5%), likely due to the inclusion of only three Ta samples, two of which arose in the renal pelvis. MIP screening for mutation and copy number change has recently been reported for multiple other cancer types [23]–[27].

We choose to focus on copy number amplifications, and identified 9 genomic regions of common amplification in an initial cohort of 80 urothelial carcinoma specimens, of which 73 gave reliable copy number information by MIP analysis. We then generated a set of MLPA probes to interrogate those nine regions in a validation cohort of 84 samples. We demonstrated that the performance of the MLPA analysis was robust on control blood DNAs, bladder cancer cell line DNAs, and frozen DNA samples from a subset (39) of the cancers initially evaluated by MIP analysis of FFPE DNA. We then performed the MLPA analysis on a separate validation cohort of 84 bladder cancer FFPE DNA samples. In the validation cohort, we found that all genomic regions showed evidence of amplification in two or more samples, with the highest levels of amplification seen for CCND1 and MDM2 (Table 6). The regions with the most frequent amplification were chromosome 1q23.3, E2F3-SOX4, and CCND1 (Table 6). Amplification was seen significantly more frequently in advanced stage tumors (Ta grade 3, or higher stage) than in early stage tumors (Ta grade 1 or 1–2).

Many previous studies have analyzed genomic copy number changes in bladder cancer using comparative genomic hybridization (CGH) or array CGH [3], [9], [10], [12], [13], [28]–[40]. All of the 9 genomic regions with amplification identified in our MIP analysis that contained genes, were highly statistically significant, and were identified in multiple samples, had been identified in these previous studies. However, MYCL1 and POLB have been identified previously in only one or two studies [9]–[11], [13], [38], [41]. In our validation cohort, we identified MYCL1 and POLB amplification in 3 and 4 samples, respectively. The validation we performed using MLPA analysis of the original MIP samples, and the consistency of these findings strongly support both the value of MIP analysis as a technology for copy number alteration detection, and provide further validation for the importance of these amplification events in bladder cancer development.

Many previous studies have also found that there is a major difference in genomic events seen in superficial papillary bladder cancer (Ta), in comparison to more advanced stages of disease. Non-invasive Ta papillary tumors commonly have activating mutations in FGFR3 (as seen here), or mutation in one of the RAS genes (mutually exclusive with FGFR3 mutation), and loss of one chromosome 9 [41]. However, few other genomic alterations have been seen by past CGH, array-CGH, or SNP analyses [11], [13], [41]. Amplification events are generally rare [41], but have recently been identified at a low level in Ta disease [36]. In contrast, many genetic events have been identified in muscle-invasive bladder cancer, including common deletion and mutations in TSC1, PTEN, RB1, and particularly TP53. In addition, recent studies have emphasized the common involvement of the PI3K–mTOR pathway in this disease [15], [42], and common mutation in chromatin remodeling genes in invasive bladder cancer [43]. Further investigation of FBXW7 in bladder cancer pathogenesis given its reported involvement in this pathway [44] is of interest.

In aggregate, these observations suggest that a relatively simple assay for amplification of these 9 genomic regions might provide useful clinical information. This might be achieved by the MLPA technique as shown here which can be performed on FFPE tissues. There are also other efficient approaches that could be used for this purpose, including digital droplet PCR [45]. However, prior to clinical use of this analysis, a similar copy number analysis will need to be performed on a large set of stage 1 bladder cancer patients in whom there is good follow-up data to assess the potential prognostic value of this assay.

Most previous genomic studies on bladder cancer have used fresh frozen tissue obtained by cystectomy. In contrast, FFPE samples are the common pathologic resource available in routine clinical practice at the time of critical decision-making. Therefore, molecular tests that can use FFPE DNA are essential. In our study, we demonstrated that MIP technology can be used to study routine FFPE cancer specimens, providing a great deal of mutational and copy number information. We also demonstrated that the simpler MLPA technique also works well on FFPE DNA.

Several of the genes included in our MLPA assay represent potential druggable targets. CCND1 amplified bladder cancers may be sensitive to CDK4 inhibitors [46]; ERBB2 amplified tumors may be sensitive to lapatinib, trastuzumab, or T-DM1 [47]; and MDM2 inhibitors are in current clinical development [48]. The specific clinical importance of each of these alterations in bladder cancer has yet to be determined, but clearly there is promise. Hence detection of amplification in these genes in bladder cancer might also lead to targeted therapy.

## Supporting Information

Figure S1 **Visualization of copy number and allele specific intensity for 330,000 SNPs using Nexus v6.0.** In each quadrant of this figure there are graphs of the intensity of signal for each pair of SNP probes (upper), and each allele (lower). Graphs are shown for four samples: a normal bladder FFPE sample in the upper left; and three different urothelial cancer FFPE samples in the other 3 quadrants. Note that the copy number graph has been normalized such that a y axis value of 0 corresponds to the normal two copies, and other values reflect either copy number loss (negative) or gain (positive). The allele fraction graph shows the relative signal intensity for each of the two alleles, and SNPs for which one allele has no signal have been screened out. The normal control bladder sample is diploid across the entire genome, and has a uniform 50% intensity value for all heterozygous SNPs. The urothelial carcinoma specimens show a variety of copy number changes and allele ratio distortions. Note region of major amplification seen on chromosomes 16p, 18p, and 21 in sample 30 at lower left, indicated by red stars.
(TIF)

Figure S2 **Amplified genomic regions in urothelial carcinoma visualized using Nexus v6.0.** In each quadrant of this figure, graphs are shown for four different urothelial carcinoma samples and for three different genomic regions. In each quadrant, chromosome cytoband is shown at top, followed by a graph of the total SNP probe intensity, then a graph of the allele specific SNP probe intensity, and then information about the nt start and end position of the amplification, the number of SNP probes in the amplification, and the probe mean and median signals within the amplification. Each dot represents a different SNP analyzed.
(TIF)

Figure S3 **MLPA analysis on control, bladder cancer cell line, and urothelial carcinoma DNA samples.** Elution intensity curves are shown for MLPA products analyzed on the ABI 3130. Y axis is light intensity in arbitrary units, reflecting fluorescence; X axis is the size of the DNA fragment being eluted from the capillary. Boxed labels indicate the gene or genomic locus for each elution peak. Note the relatively even size of all probe peaks in the control sample. Note that there is selective increase in the relative signals for the SOX4 and E2F3 probes in the bladder cancer cell line sample. Note that other regions of relative increase are seen in the lower two urothelial carcinoma samples, E2F3 and POLB, respectively.
(TIF)

Table S1 **List of all urothelial carcinoma samples analyzed.**
(XLS)

Table S2 **List of all mutations assessed in the MIP analysis.**
(XLSX)

Table S3 **Chromosomal regions identified by GISTIC analysis of MIP data with significant CN gains or losses, with q<0.1.** The chromosomal region, CN change, q value, and G-score (GISTIC) are shown for each region.
(XLS)

Table S4 **MLPA probe sequences used in this study.**
(XLSX)
