# Supplementary figures and images for: Identification of Nine Genomic Regions of Amplification in Urothelial Carcinoma, Correlation with Stage, and Potential Prognostic and Therapeutic Value
Source: PLoS One. 2013 Apr 4;8(4):e60927. doi: 10.1371/journal.pone.0060927 (PMC3617176; doi:10.1371/journal.pone.0060927)

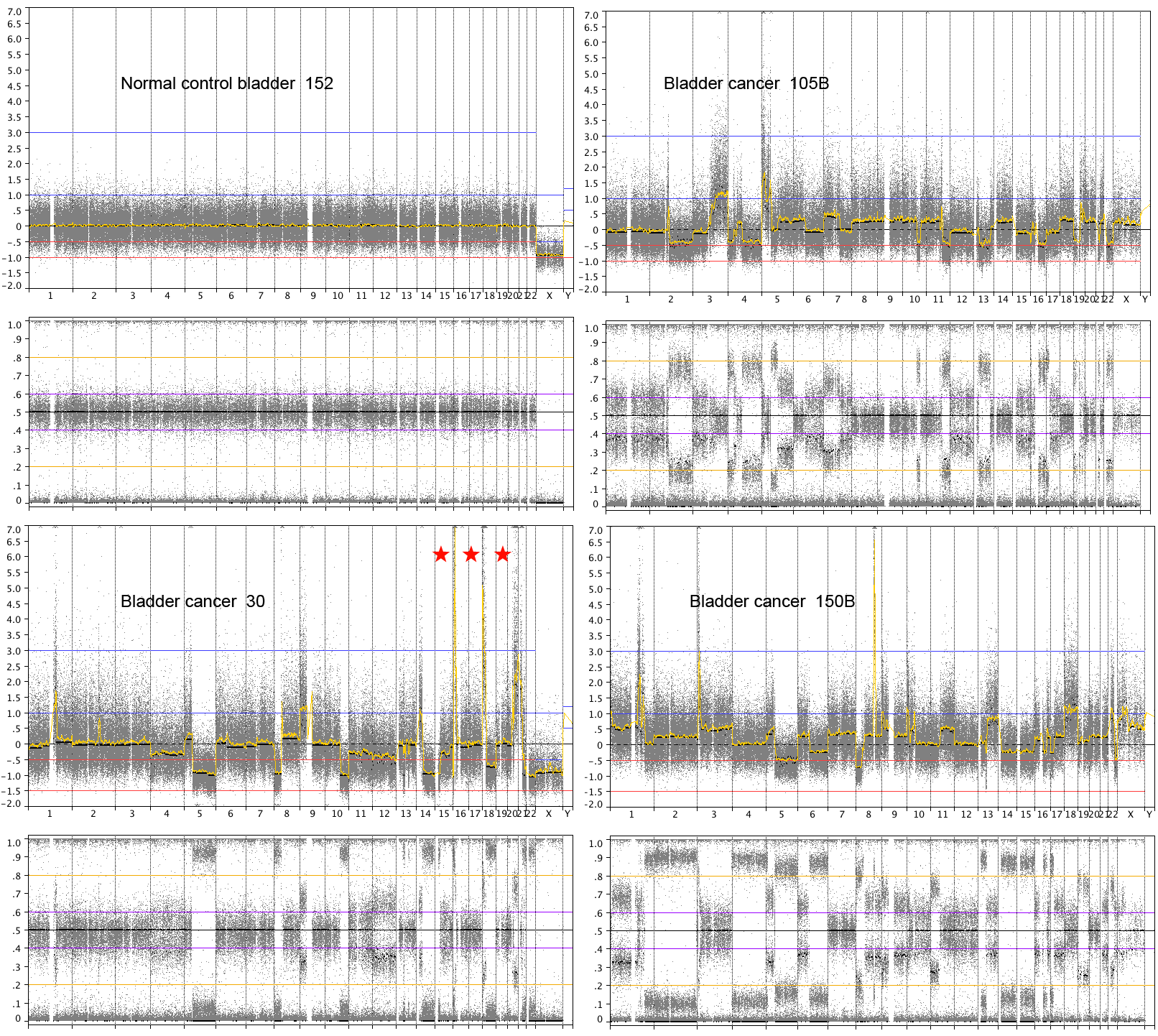

Supplement: Figure S1 — Visualization of copy number and allele specific intensity for 330,000 SNPs using Nexus v6.0. In each quadrant of this figure there are graphs of the intensity of signal for each pair of SNP probes (upper), and each allele (lower). Graphs are shown for four samples: a normal bladder FFPE sample in the upper left; and three different urothelial cancer FFPE samples in the other 3 quadrants. Note that the copy number graph has been normalized such that a y axis value of 0 corresponds to the normal two copies, and other values reflect either copy number loss (negative) or gain (positive). The allele fraction graph shows the relative signal intensity for each of the two alleles, and SNPs for which one allele has no signal have been screened out. The normal control bladder sample is diploid across the entire genome, and has a uniform 50% intensity value for all heterozygous SNPs. The urothelial carcinoma specimens show a variety of copy number changes and allele ratio distortions. Note region of major amplification seen on chromosomes 16p, 18p, and 21 in sample 30 at lower left, indicated by red stars. (TIF) [file pone.0060927.s001.tif]

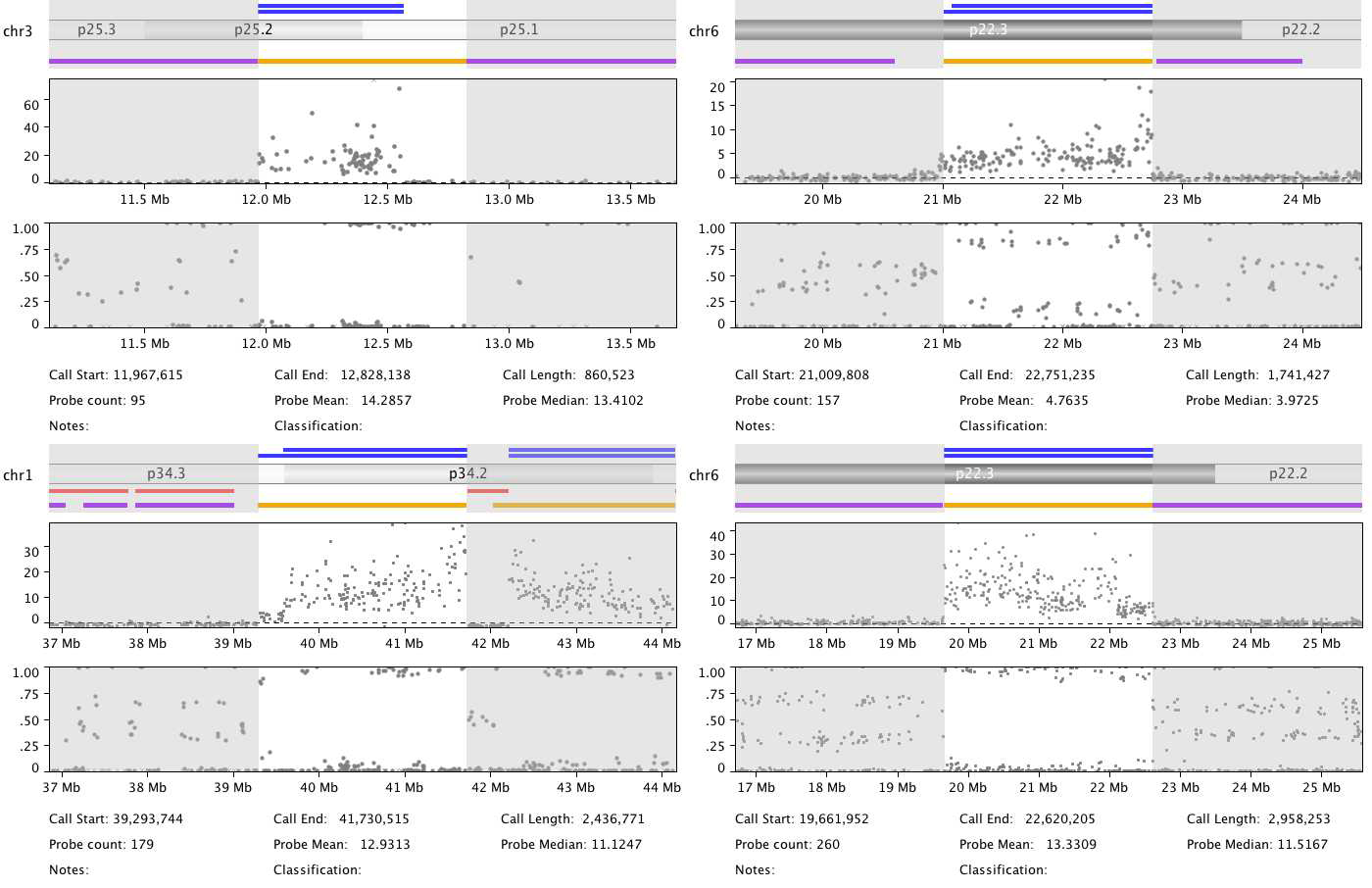

Supplement: Figure S2 — Amplified genomic regions in urothelial carcinoma visualized using Nexus v6.0. In each quadrant of this figure, graphs are shown for four different urothelial carcinoma samples and for three different genomic regions. In each quadrant, chromosome cytoband is shown at top, followed by a graph of the total SNP probe intensity, then a graph of the allele specific SNP probe intensity, and then information about the nt start and end position of the amplification, the number of SNP probes in the amplification, and the probe mean and median signals within the amplification. Each dot represents a different SNP analyzed. (TIF) [file pone.0060927.s002.tif]

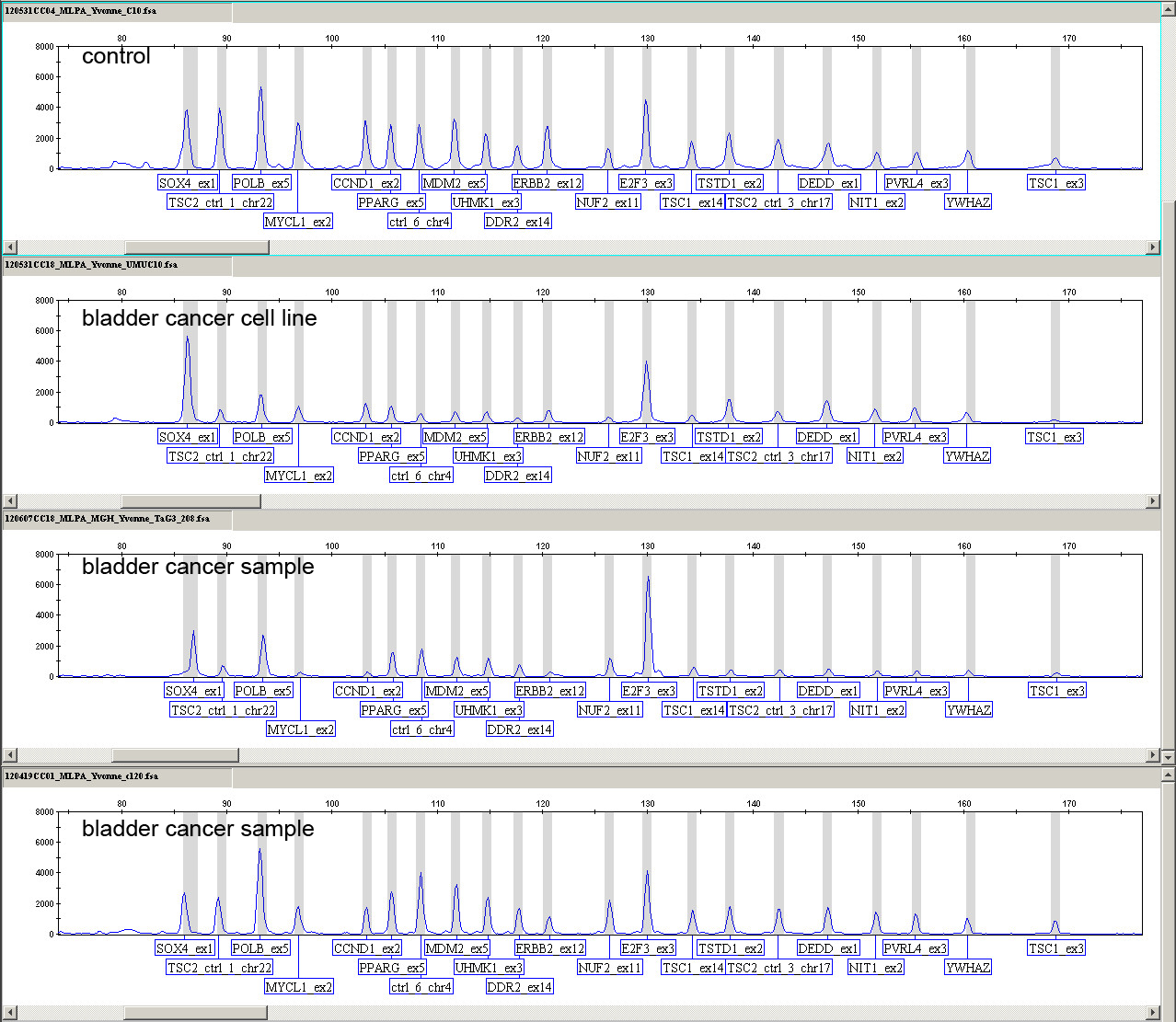

Supplement: Figure S3 — MLPA analysis on control, bladder cancer cell line, and urothelial carcinoma DNA samples. Elution intensity curves are shown for MLPA products analyzed on the ABI 3130. Y axis is light intensity in arbitrary units, reflecting fluorescence; X axis is the size of the DNA fragment being eluted from the capillary. Boxed labels indicate the gene or genomic locus for each elution peak. Note the relatively even size of all probe peaks in the control sample. Note that there is selective increase in the relative signals for the SOX4 and E2F3 probes in the bladder cancer cell line sample. Note that other regions of relative increase are seen in the lower two urothelial carcinoma samples, E2F3 and POLB, respectively. (TIF) [file pone.0060927.s003.tif]
